# Association between the glucose-lipid metabolism 7-factor index and hyperuricemia in patients with type 2 diabetes: a retrospective study

**DOI:** 10.3389/fendo.2026.1961640

**Published:** 2026-10-21

**Authors:** Zhangna Zheng, Qianyi Li, Huijuan Zhang

**Affiliations:** Department of Endocrinology, The First Affiliated Hospital of Harbin Medical University, Harbin, China

**Keywords:** GLM7, glucose-lipid metabolism 7-factor index, hyperuricemia, insulin resistance, serum uric acid, type 2 diabetes

## Abstract

**Background:**

The glucose-lipid metabolism 7-factor index (GLM7) is a composite metabolic index that integrates age, body mass index, fasting blood glucose, insulin, triglycerides, low-density lipoprotein cholesterol, and high-density lipoprotein cholesterol. However, the specific correlation between this index and hyperuricemia (HUA) in patients with type 2 diabetes (T2DM) has not been clarified to date. This study investigated the association between GLM7 and HUA, and evaluated the discriminatory performance of this index in hospitalized patients with T2DM.

**Methods:**

This retrospective cross-sectional study enrolled 959 patients with T2DM admitted to the First Affiliated Hospital of Harbin Medical University between January and December 2024. GLM7 was calculated based on routinely collected clinical and biochemical indicators. HUA was defined in accordance with established serum uric acid (SUA) criteria. Multivariable logistic regression and linear regression models were constructed respectively to analyze the associations of GLM7 with HUA and SUA. Restricted cubic spline (RCS) analysis was conducted to identify the specific pattern of the association. Receiver operating characteristic (ROC) curve analysis was applied to evaluate the discriminatory performance of GLM7. Incremental discriminatory capacity and model fitting were further compared between the conventional clinical model with and without the inclusion of GLM7.

**Results:**

Of the 959 enrolled patients, 259 (27.0%) were diagnosed with HUA. The GLM7 level was significantly higher in patients with HUA compared with those without HUA. After multivariable adjustment, elevated GLM7 was independently associated with increased odds of HUA (OR per 1-unit increase: 1.72; 95% CI: 1.29–2.28; *P* < 0.001). Compared with patients in the lowest GLM7 quartile, those in the highest quartile had significantly higher odds of HUA (OR: 2.04; 95% CI: 1.28–3.26; *P* = 0.003). Elevated GLM7 was also significantly associated with increased SUA concentrations, with an adjusted increment of 37.88 μmol/L (95% CI: 28.22–47.55; *P* < 0.001) per 1-unit increase in GLM7. Restricted cubic spline analysis confirmed a significant overall association between GLM7 and HUA, and no statistically significant nonlinear association was detected. GLM7 exhibited limited discriminatory performance for HUA, with an area under the ROC curve of 0.599 (95% CI: 0.559–0.640). The optimal cutoff value was 7.959, with a sensitivity of 63.3% and a specificity of 55.0%. Adding GLM7 to the conventional clinical model did not significantly improve the area under the ROC curve (0.754 vs 0.757; DeLong *P* = 0.438), while model fitting was significantly improved (likelihood-ratio χ²=5.70, *P* = 0.017).

**Conclusions:**

GLM7 is independently and positively associated with HUA and SUA levels among hospitalized patients with T2DM. Nevertheless, GLM7 exhibits limited standalone discriminatory capacity, and its addition to the conventional clinical model does not yield a significant improvement in discriminatory performance. The present findings support the role of GLM7 as a composite metabolic marker correlated with hyperuricemia in patients with T2DM, but do not validate its use as a diagnostic or screening tool.

## 1 Introduction

Hyperuricemia (HUA), defined by elevated serum uric acid (SUA) concentration, is an increasingly prevalent metabolic abnormality and a major public health concern. In China, nationally representative surveys indicate that the prevalence of HUA among adults increased from 11.1% in 2015–2016 to 14.0% in 2018–2019. HUA is closely associated with metabolic dysregulation and frequently coexists with multiple cardiometabolic diseases, in particular obesity, hypertension, dyslipidemia, and impaired renal function (1–10).Therefore, identifying metabolic characteristics associated with elevated SUA may help improve the understanding and risk stratification of HUA in high-risk populations.

HUA and type 2 diabetes (T2DM) frequently coexist and share multiple metabolic characteristics, particularly obesity, insulin resistance, and dyslipidemia. More importantly, the two conditions exhibit a bidirectional association at both the epidemiological and metabolic levels. Prospective cohort studies and meta-analyses have consistently demonstrated that higher SUA concentrations are correlated with an increased subsequent risk of T2DM. For instance, a meta-analysis of prospective cohort studies enrolling over 32,000 participants revealed that individuals with the highest SUA levels faced a higher risk of developing T2DM compared with those with the lowest levels, and this association remained statistically significant after adjustment for established metabolic risk factors (11). Consistent findings have also been reported in a prospective Chinese cohort, in which higher baseline SUA levels and longitudinal increases in SUA were associated with the subsequent development of T2DM (12). Conversely, metabolic disturbances accompanying T2DM, particularly insulin resistance and hyperinsulinemia, may affect renal uric acid handling and contribute to elevated SUA levels (13). Accordingly, the co-occurrence of HUA and T2DM may partially reflect shared metabolic abnormalities, rather than an independent perturbation of glucose or uric acid metabolism alone.

Given the close interconnections between glucose and lipid dysregulation, insulin resistance, and uric acid metabolism, composite metabolic indicators have garnered growing attention as practical biomarkers for reflecting overall metabolic status. The triglyceride-glucose (TyG) index is a representative marker of this category and has been extensively studied as a surrogate indicator of insulin resistance. More recently, the glucose-lipid metabolism 7-factor index (GLM7) was constructed based on routine clinical indicators from the National Health and Nutrition Examination Survey (NHANES). GLM7 integrates the information of age, body mass index (BMI), fasting blood glucose (FBG), insulin, triglycerides (TG), low-density lipoprotein cholesterol (LDL-C), and high-density lipoprotein cholesterol (HDL-C), and thus provides a comprehensive characterization of multiple dimensions of glucose and lipid metabolism (14). The original study reported correlations between GLM7 and multiple metabolic diseases, and validated its predictive performance in independent populations.

However, it remains unclear whether GLM7 is correlated with HUA in patients diagnosed with T2DM This question necessitates targeted investigation: fasting blood glucose is not only a core component of GLM7, but also a key diagnostic indicator for diabetes mellitus. Accordingly, the clinical value of GLM7 in the diabetic population requires independent verification, rather than direct extrapolation from studies conducted in the general population. In addition, since GLM7 was initially constructed based on NHANES data, its applicability in Chinese T2DM patients still requires further confirmation.

Therefore, the present study aimed to investigate the correlation between GLM7 and HUA as well as SUA levels in hospitalized T2DM patients, and verify the robustness and clinical relevance of this correlation through multivariable regression analysis, dose-response analysis, subgroup analysis and receiver operating characteristic analysis.

## 2 Materials and methods

### 2.1 Study cohort

A total of 959 patients with type 2 diabetes admitted to the Department of Endocrinology, the First Affiliated Hospital of Harbin Medical University between January 2024 and December 2024 were retrospectively enrolled and analyzed in this study. The flow chart of study participant screening and enrollment is presented in **Figure 1**. This study was approved by the Ethics Committee of the First Affiliated Hospital of Harbin Medical University (Approval No. 2026385), and all research procedures were implemented in accordance with the Declaration of Helsinki and its subsequent revisions.

**Figure 1:**
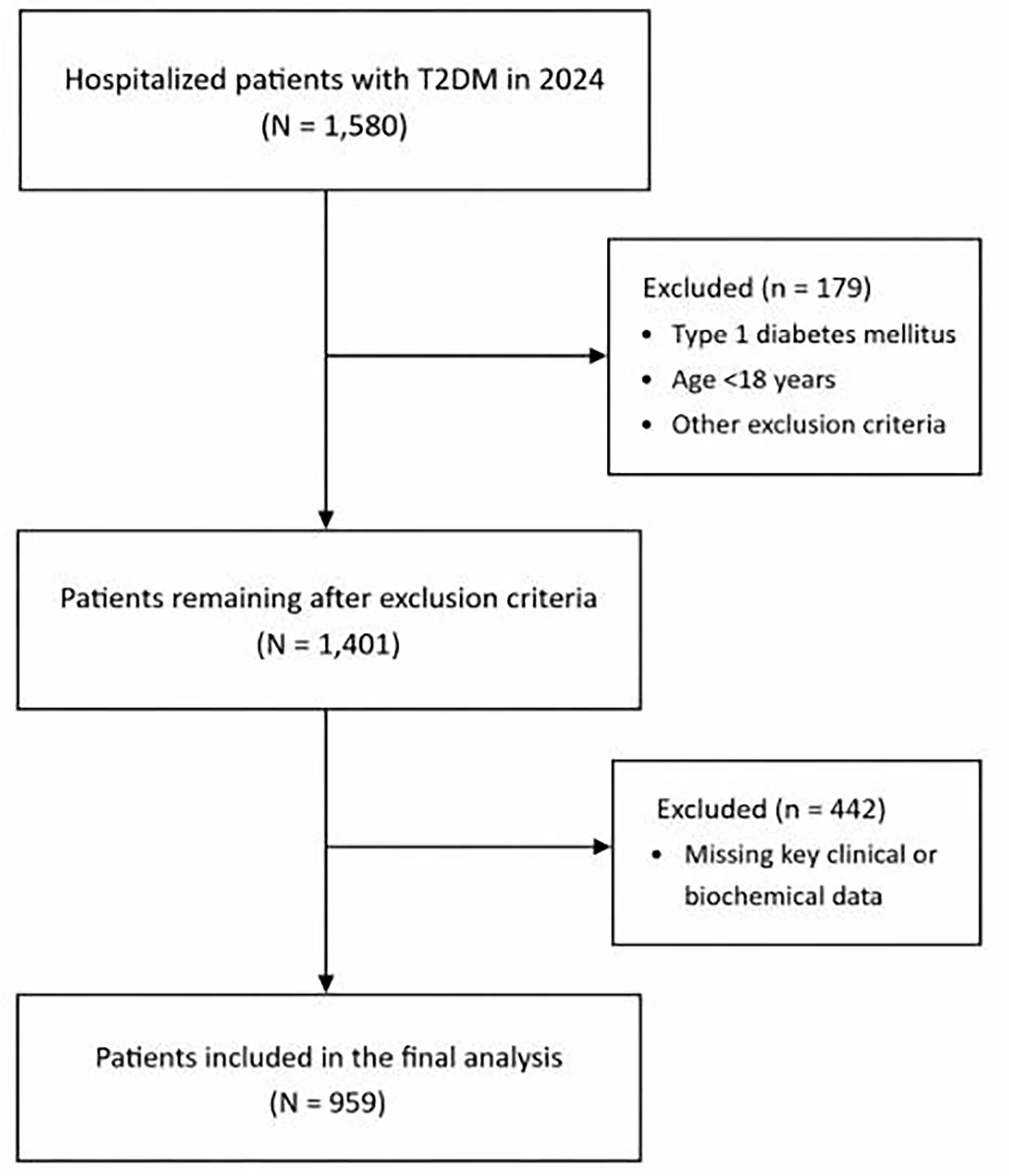
Flow diagram of patient selection.

#### 2.1.1 Inclusion criteria

Patients with type 2 diabetes who met the diagnostic criteria of the Guideline for the Prevention and Treatment of Type 2 Diabetes in China (2024 Edition) (15): (1) typical symptoms with random blood glucose ≥11.1 mmol/L; (2) fasting blood glucose ≥7.0 mmol/L; (3) 2-hour plasma glucose during an oral glucose tolerance test ≥11.1 mmol/L; (4) HbA1c ≥6.5%; (5) patients without typical diabetic symptoms required confirmation by repeat testing on a separate day. A diagnosis of diabetes was established if any one of the above criteria was met; and patients were required to have complete biochemical and clinical data.

#### 2.1.2 Exclusion criteria

(1) Type 1 diabetes mellitus (T1DM), gestational diabetes mellitus, and other special types of diabetes; (2) Age <18 years; (3) Incomplete baseline data; (4) Use of exogenous insulin or lipid-lowering medications prior to admission; (5)application of urate-lowering agents;(6) Pancreatitis or post-pancreatic surgery; (7) Stress states such as diabetic ketoacidosis or hyperosmolar hyperglycemic syndrome; (8) Malignancy-related diseases; (9) Presence of other types of kidney disease, such as primary glomerulonephritis, or renal impairment related to infectious disease, drug use, pregnancy, vascular disease, urinary tract disease, or hereditary disease, or patients undergoing renal replacement therapy, including dialysis and kidney transplantation.

### 2.2 Data collection

General demographic characteristics were collected, including age, sex, duration of diabetes, history of hypertension, as well as smoking and drinking status. Smoking was defined as having consumed more than 1 cigarette per day for at least 1 consecutive year and maintaining the smoking habit at the time of enrollment; alcohol consumption was defined as having consumed alcoholic beverages including liquor, beer or wine at least 2 days per week for no less than 1 consecutive year.

Physical examination indicators were collected, including systolic blood pressure (SBP), diastolic blood pressure (DBP), body height and body weight, and body mass index (BMI) was calculated based on the obtained height and weight data.

Information on the current hypoglycemic regimen for type 2 diabetes and the patients’ current medication usage was also recorded.

Laboratory tests and examinations, including HbA1c (%), serum C-peptide (ng/mL), serum insulin (μU/mL), alanine aminotransferase (ALT, U/L), aspartate aminotransferase (AST, U/L), total cholesterol (TC, mmol/L), triglycerides (TG, mmol/L), low-density lipoprotein cholesterol (LDL-C, mmol/L), high-density lipoprotein cholesterol (HDL-C, mmol/L), creatinine (SCr, μmol/L), blood urea nitrogen (BUN, mmol/L), and serum uric acid (SUA, μmol/L).

### 2.3 Calculation of GLM7 and definition of hyperuricemia

The GLM7 index was calculated using the following formula, as previously described (14):

GLM7 = log_10_(Age (years) × BMI (kg/m²) × FBG (mg/dL) × Insulin (pmol/L) × TG (mmol/L) × LDL-c (mmol/L)/HDL-c (mmol/L)).

The TyG index was calculated as follows (16):

TyG index = ln [TG (mg/dL) × FPG (mg/dL)/2].

Based on the physiological saturation threshold of uric acid, hyperuricemia (HUA) was diagnosed according to the following criteria: a serum uric acid concentration ≥360 μmol/L (6.0 mg/dL) in women, and ≥420 μmol/L (7.0 mg/dL) in men (17).

### 2.4 Statistical analysis

All statistical analyses in this study were performed using R software (version 4.4.3). The following R packages were used: rms (8.1-0) for multivariable logistic regression and restricted cubic spline analysis; pROC (1.19.0.1) for ROC curve analysis and DeLong tests; car (3.1-5) for variance inflation factor calculation; lmtest (0.9-40) for likelihood-ratio tests; tableone (0.13.2) and gtsummary (2.5.1) for descriptive tables; shapviz (0.10.3) for SHAP value visualization; ggplot2 (4.0.3), forestplot (3.1.7), patchwork (1.3.2), and ggpubr (0.6.2) for data visualization; and readxl (1.4.5), dplyr (1.2.1), tidyr (1.3.2), janitor (2.2.1), and broom (1.0.13) for data manipulation and tidying. For continuous variables, normally distributed data are reported as the mean ± standard deviation (SD), while non-normally distributed data are expressed as the median (interquartile range [IQR]). For between-group comparisons of the hyperuricemia group and the non-hyperuricemia group, the independent-samples t-test was adopted for normally distributed continuous variables, and the Mann–Whitney U test was applied for non-normally distributed continuous variables. Categorical variables are described as counts (percentages), and between-group comparisons were performed using the chi-square test or Fisher’s exact test based on actual conditions. Multivariable logistic regression analysis was employed to examine the association between GLM7 and the prevalence of hyperuricemia, with the results reported as odds ratios (ORs) with corresponding 95% confidence intervals (CIs). Furthermore, linear regression analysis was conducted to investigate the association between GLM7 and serum uric acid (SUA) levels. To control for potential confounding factors, sequentially adjusted regression models were established. Based on the fitted multivariable logistic regression model, the SHapley Additive exPlanations (SHAP) approach was applied to quantify and visualize the contribution of each variable to the prediction of hyperuricemia.To further characterize the dose-response relationship between GLM7 and hyperuricemia, restricted cubic spline (RCS) analysis was conducted, with four knots located at the 5th, 35th, 65th, and 95th percentiles of the GLM7 distribution. This setting balances sufficient flexibility for fitting potential non-linear associations and controls the complexity of the model. The overall association and the deviation from linearity were tested separately.

Subgroup analyses stratified by age, sex, BMI, hypertension, HbA1c, smoking status, and alcohol consumption were performed to evaluate the robustness of the observed association, and interaction tests were applied to examine effect heterogeneity across subgroups. Receiver operating characteristic (ROC) curve analysis was adopted to assess the discriminative performance of GLM7 for hyperuricemia. To detect potential multicollinearity among covariates, variance inflation factors (VIFs) were calculated for all variables included in the fully adjusted model, and a VIF value less than 5 was defined as the absence of significant multicollinearity.

Given that serum creatinine (SCr) and estimated glomerular filtration rate (eGFR) are two highly correlated indicators reflecting renal function, additional sensitivity analyses were conducted by including SCr or eGFR separately in the multivariable model. The robustness of the association between GLM7 and hyperuricemia was verified by comparing effect estimates across different models. All statistical tests were two-sided*, and P* < 0.05 was considered to indicate a statistically significant difference.

## 3 Results

### 3.1 Baseline characteristics

A total of 959 patients with type 2 diabetes were enrolled in this study, of whom 259 (27.0%) were diagnosed with hyperuricemia, and 700 (73.0%) did not have hyperuricemia (**Table 1**). Compared with the non-hyperuricemia group, patients in the hyperuricemia group were younger (*P* < 0.001), and had significantly higher DBP, TC, TG, SCr, fasting insulin levels, and higher BMI, while their eGFR was significantly lower (all *P* < 0.001). Additionally, AST levels in the hyperuricemia group were slightly higher than those in the non-hyperuricemia group (*P* = 0.026). No statistically significant between-group differences were found in sex, smoking status, alcohol consumption status, hypertension prevalence, diabetes duration, SBP, FBG, HbA1c, HDL-C, LDL-C, or ALT (all *P*>0.05). Notably, GLM7 levels in patients with hyperuricemia were significantly higher than those in patients without hyperuricemia (*P* < 0.001).

**Table 1:** Baseline characteristics by hyperuricemia status.

Characteristic	Total (n = 959)	Non-Hyperuricemia (n = 700)	Hyperuricemia (n = 259)	*P*
Age(years)	57.00 (48.50, 64.00)	58.00 (50.00, 65.00)	54.00 (45.00, 63.00)	<.001
Male, n(%)	578 (60.27)	431 (61.57)	147 (56.76)	0.176
Smoke, n(%)	289 (30.14)	221 (31.57)	68 (26.25)	0.111
Drink, n(%)	294 (30.66)	220 (31.43)	74 (28.57)	0.394
Hypertension, n(%)	433 (45.15)	308 (44.00)	125 (48.26)	0.239
Diabetes Duration(years)	10.00 (5.00, 16.00)	10.00 (6.00, 17.00)	10.00 (5.00, 16.00)	0.094
SBP(mmHg)	136.00(123.00,151.00)	135.00(122.00, 151.25)	137.00(124.50,151.00)	0.222
DBP(mmHg)	85.00 (78.00, 94.00)	84.00 (77.00, 92.25)	88.00 (80.00, 96.00)	<.001
FBG(mmol/L)	8.44 (6.84, 10.86)	8.45 (6.79, 10.93)	8.38 (7.08, 10.52)	0.778
HbA1C(%)	8.40 (7.30, 9.70)	8.40 (7.40, 9.80)	8.30 (7.25, 9.70)	0.593
TC(mmol/L)	4.85 (4.04, 5.81)	4.76 (3.99, 5.68)	5.12 (4.20, 6.12)	0.003
TG(mmol/L)	1.73 (1.19, 2.76)	1.60 (1.13, 2.55)	2.12 (1.44, 3.54)	<.001
HDL-C(mmol/L)	1.13 (0.95, 1.31)	1.13 (0.95, 1.33)	1.12 (0.95, 1.29)	0.591
LDL-C(mmol/L)	2.96 (2.35, 3.65)	2.94 (2.36, 3.58)	3.03 (2.34, 3.88)	0.069
SCr(μmol/L)	65.00 (55.00, 77.00)	63.30 (53.30, 73.00)	73.10 (60.00, 87.10)	<.001
Insulin(μU/mL)	11.77 (8.07, 16.82)	11.16 (7.72, 16.29)	13.30 (9.04, 18.58)	<.001
ALT(U/L)	20.70 (14.95, 31.90)	20.30 (15.07, 31.10)	22.30 (14.85, 35.00)	0.089
AST(U/L)	19.60 (15.80, 25.30)	19.40 (15.60, 24.92)	20.30 (16.10, 27.95)	0.026
eGFR(mL/min/1.73m^2^)	103.13(93.58, 110.75)	104.00 (95.70, 111.00)	98.93 (76.51, 109.95)	<.001
BMI(kg/m^2^)	25.46 (23.51, 27.73)	25.27 (23.36, 27.34)	26.33 (24.30, 29.23)	<.001
GLM7	7.96 (7.57, 8.34)	7.89 (7.53, 8.28)	8.13 (7.72, 8.50)	<.001

### 3.2 Association between GLM7 and hyperuricemia

**Table 2** summarizes the results of multivariable logistic regression analysis examining the association between the GLM7 index and hyperuricemia. In Model 1 without adjustment for confounding variables, every 1-unit increment in the GLM7 index was significantly associated with higher odds of hyperuricemia (OR = 1.83, 95% CI: 1.42–2.36, P<0.001). This association remained robust and statistically significant in Model 2 (adjusted for sex) and Model 3 (further adjusted for smoking status, alcohol consumption, hypertension, HbA1c, SCr, ALT, AST, and eGFR; Model 3: OR = 1.72, 95% CI: 1.29–2.28, P<0.001). When the GLM7 index was stratified into quartiles, with the lowest quartile (Q1) set as the reference, the Q3 and Q4 groups exhibited significantly higher odds of hyperuricemia in the fully adjusted model, with OR values of 1.78 (95% CI: 1.13–2.83, P = 0.014) and 2.04 (95% CI: 1.28–3.26, P = 0.003) respectively, indicating a significant dose-response relationship (P<0.001). Additionally, every 1-SD increment in the GLM7 index was associated with higher odds of hyperuricemia (Model 3: OR = 1.38, 95% CI: 1.17–1.64, P<0.001). SHAP analysis (**Figure 2**) was further conducted to quantify the relative contribution of each variable to the model’s hyperuricemia prediction, and the results indicated that GLM7 contributed considerably to the model’s prediction of hyperuricemia.

**Table 2:** Logistic regression of GLM7 and hyperuricemia.

Hyperuricemia	OR95%CI		
	Model 1	Model 2	Model 3
GLM7	1.83 (1.42-2.36) <0.001	1.82 (1.42-2.35)<0.001	1.72 (1.29-2.28)<0.001
Each SD increase	1.44 (1.23-1.67)<0.001	1.43 (1.23-1.66)<0.001	1.38 (1.17-1.64)<0.001
Quantiles			
Quantile 1	reference	reference	reference
Quantile 2	0.92 (0.59-1.45)0.731	0.91 (0.58-1.42)0.675	0.99 (0.61-1.60)0.958
Quantile 3	1.88 (1.24-2.84)0.003	1.86 (1.23-2.82)0.003	1.78 (1.13-2.83)0.014
Quantile 4	2.19 (1.45-3.30)<0.001	2.17 (1.44-3.27)<0.001	2.04 (1.28-3.26)0.003
*P* for trend	<0.001	<0.001	<0.001

OR, odds ratio; 95% CI, 95% confidence interval; SD, standard deviation.

Model1: non-adjusted.

Model2: adjusted for sex.

Model3: adjusted for sex, smoke, drink, Hypertension, HbA1c, SCr, ALT, AST, eGFR.

**Figure 2:**
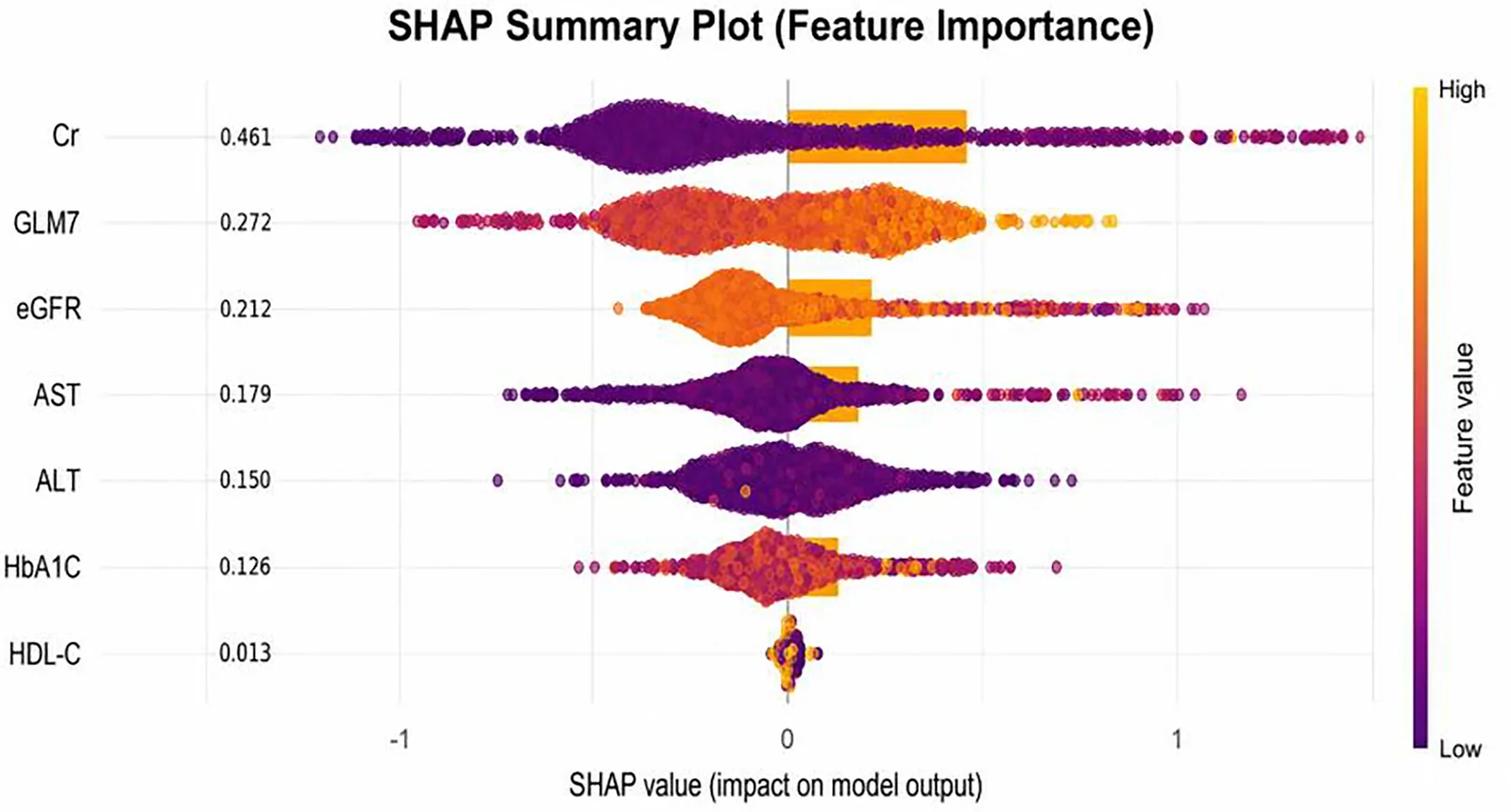
SHAP summary plot of associated features for hyperuricemia.

### 3.3 Association between GLM7 and serum uric acid

**Table 3** summarizes the results of linear regression analysis examining the association between the GLM7 index and serum uric acid (SUA) levels. In the fully adjusted model, every 1-unit increment in the GLM7 index was associated with a 37.88 μmol/L elevation in SUA levels (β=37.88, 95% CI: 28.22–47.55, *P* < 0.001). Consistent with the findings of the preceding logistic regression analysis, SUA levels increased significantly across ascending GLM7 index quartiles (*P* for trend <0.001). Relative to the lowest quartile (Q1), the highest quartile (Q4) exhibited a mean SUA elevation of 57.07 μmol/L (95% CI: 40.74–73.40, *P* < 0.001). Every 1-standard-deviation increment in the GLM7 index was also significantly associated with a 22.60 μmol/L elevation in SUA levels (95% CI: 16.83–28.36, *P* < 0.001). Restricted cubic spline (RCS) analysis confirmed a significant overall association between GLM7 and hyperuricemia (HUA) (P for overall association <0.001), and no statistically significant evidence of a non-linear association was detected (P for non-linearity = 0.656) (**Figure 3**).

**Table 3:** Linear regression of GLM7 and SUA levels.

SUA levels	β95%CI		
	Model 1	Model 2	Model 3
GLM7	38.22 (28.03-48.40)<0.001	40.51 (30.70-50.32)<0.001	37.88 (28.22-47.55)<0.001
Each SD increase	22.80 (16.72-28.87)<0.001	24.17 (18.32-30.02)<0.001	22.60 (16.83-28.36)<0.001
Quantiles			
Quantile 1	reference	reference	reference
Quantile 2	6.76 (-10.37-23.88)0.440	11.10 (-5.44-27.64)0.189	13.76 (-1.97-29.50)0.087
Quantile 3	42.21 (25.09-59.34)<0.001	44.17 (27.65-60.69)<0.001	39.45 (23.47-55.42)<0.001
Quantile 4	58.08 (40.94-75.22)<0.001	60.78 (44.24-77.32)<0.001	57.07 (40.74-73.40)<0.001
*P* for trend	<0.001	<0.001	<0.001

β, regression coefficient; 95% CI, 95% confidence interval; SD, standard deviation.

Model1: non-adjusted.

Model2: adjusted for sex.

Model3: adjusted for sex, smoke, drink, Hypertension, HbA1c, SCr, ALT, AST, eGFR.

**Figure 3:**
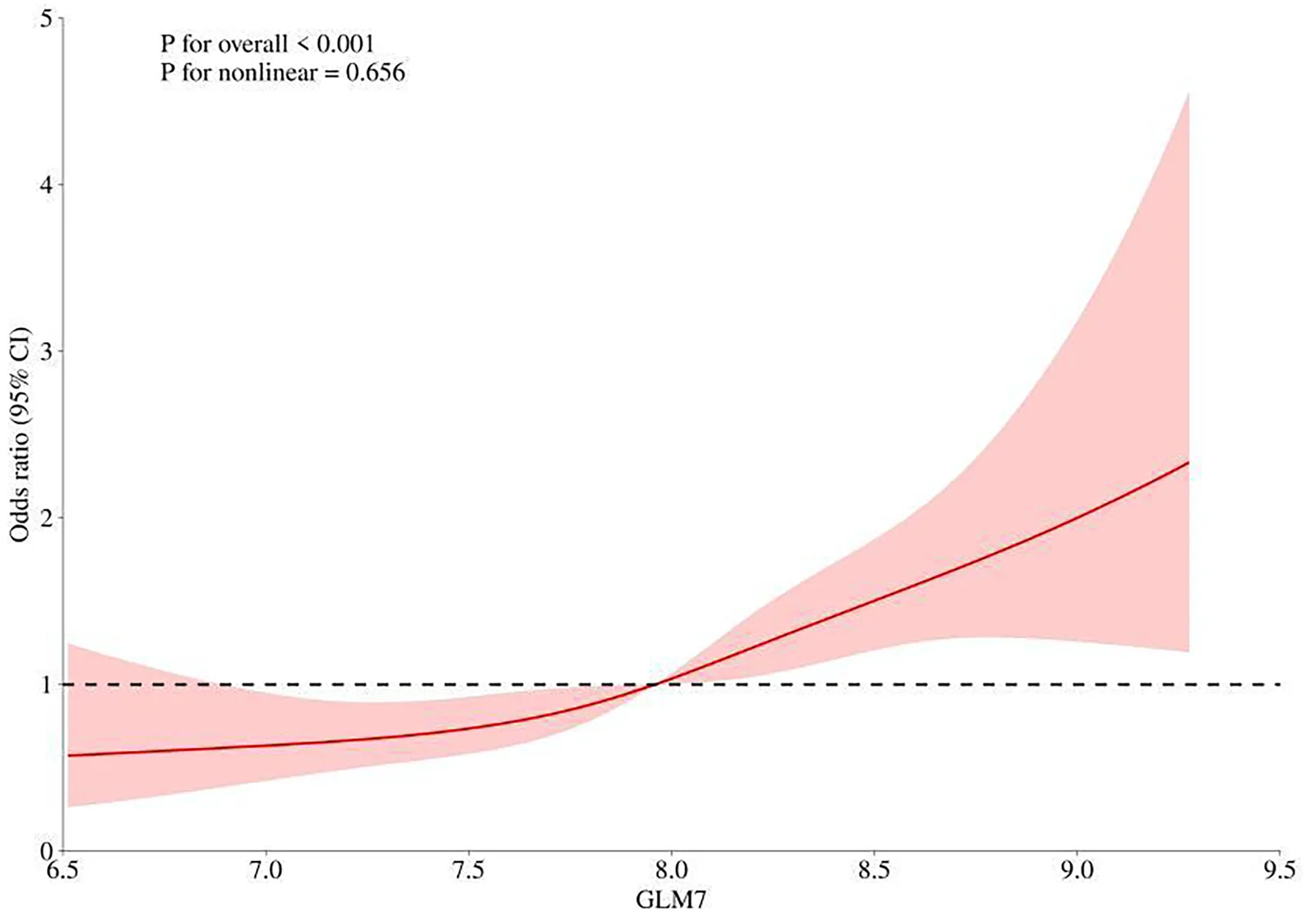
Dose-response relationship between GLM7 and hyperuricemia.

### 3.4 Subgroup analysis and ROC analysis

The results of the subgroup analyses are presented in **Figure 4**. The positive association between GLM7 and hyperuricemia remained consistent across all subgroups stratified by age, sex, smoking status, alcohol consumption status, hypertension status, and BMI. No statistically significant interaction effects were detected between subgroups (all *P* for interaction >0.05). These results indicate that the association between GLM7 and hyperuricemia is relatively stable and not substantially modified by key clinical characteristics.

**Figure 4:**
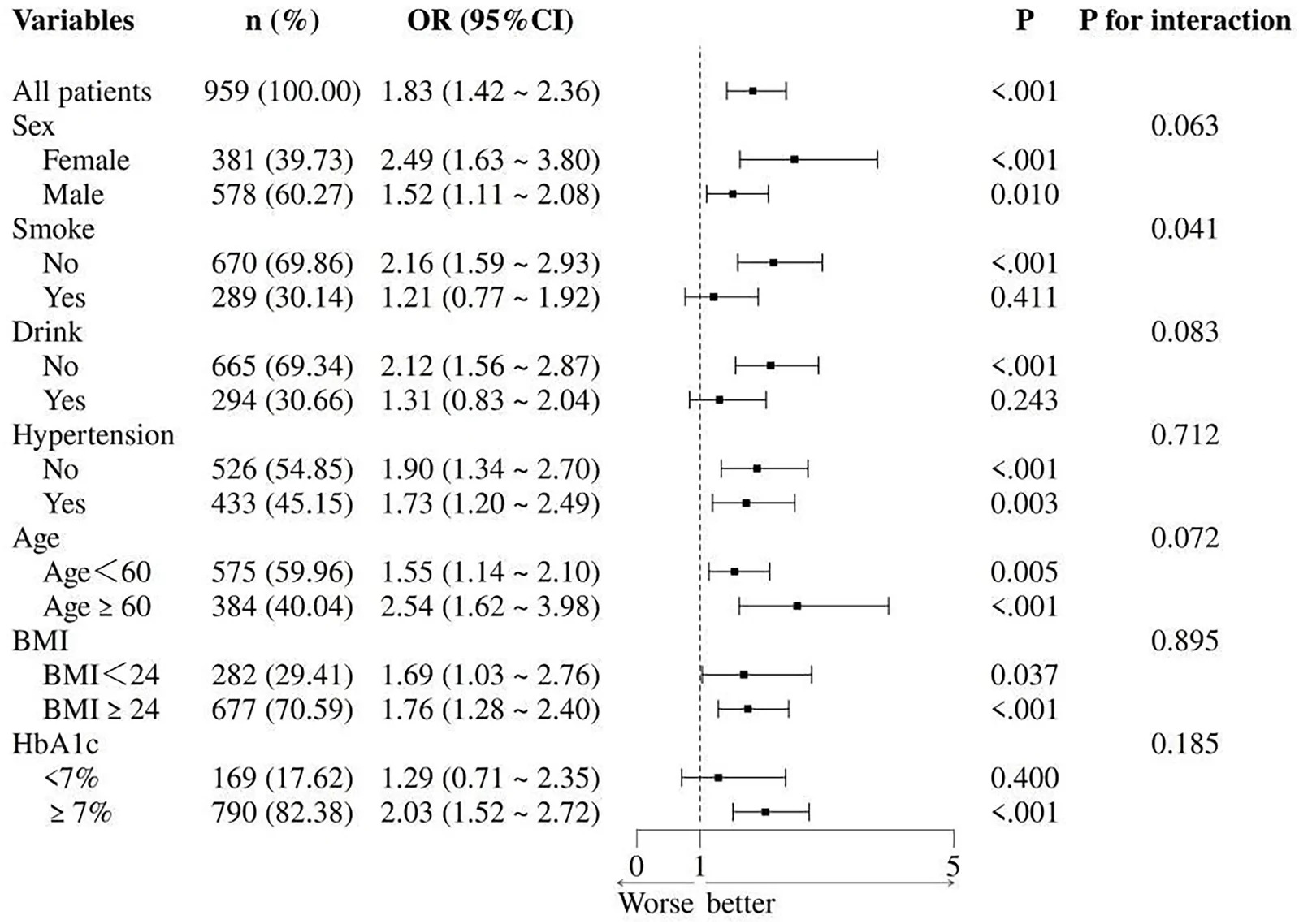
Subgroup analyses of the association between GLM7 and hyperuricemia.

Receiver operating characteristic (ROC) curve analysis was conducted to assess the discriminative performance of the GLM7 index for hyperuricemia among patients with type 2 diabetes (T2DM) (**Figure 5**). The area under the curve (AUC) for GLM7 was 0.599 (95% CI: 0.559–0.640), with the optimal cutoff value determined as 7.959, corresponding to a sensitivity of 63.3% and a specificity of 55.0%. No statistically significant difference was observed between the AUC of GLM7 and that of the triglyceride-glucose (TyG) index (AUC = 0.593, 95% CI: 0.554–0.633; DeLong test, *P* = 0.668), triglyceride (TG, AUC = 0.623, 95% CI: 0.585–0.662; *P* = 0.106), or body mass index (BMI, AUC = 0.598, 95% CI: 0.557–0.639; *P* = 0.965) (**Tables 4**, **5**). These results suggest that GLM7 exhibits limited discriminative capacity for hyperuricemia when applied as an independent biomarker.

**Figure 5:**
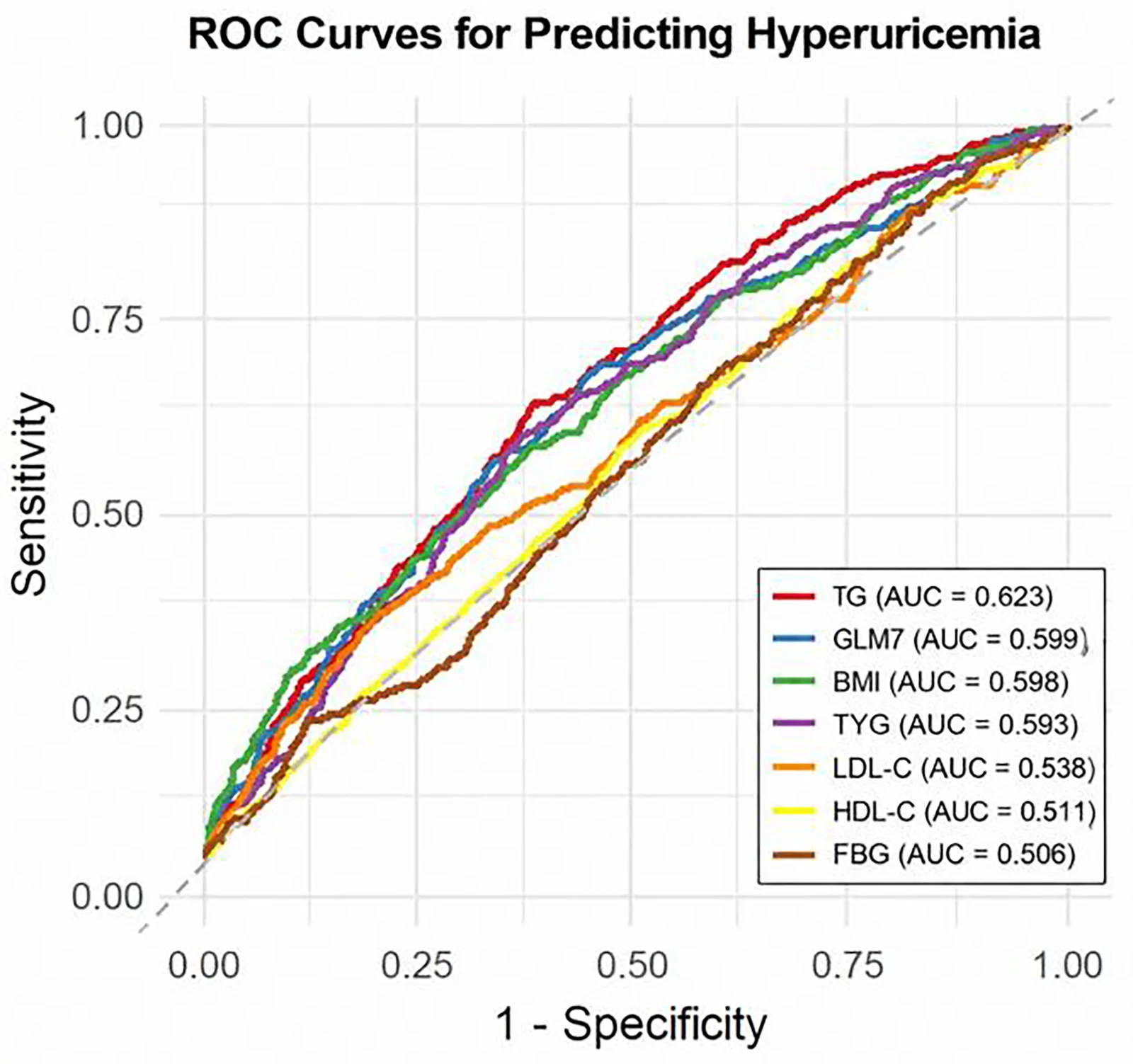
ROC curve comparison among GLM7 and other indicators for hyperuricemia.

**Table 4:** Discriminative performance of GLM7 and individual metabolic parameters for hyperuricemia.

Variable	AUC	95% CI	Cut-off	Sensitivity	Specificity
GLM7	0.599	0.559–0.640	7.959	63.3%	55.0%
TyG	0.593	0.554–0.633	9.534	56.0%	61.4%
TG	0.623	0.585–0.662	1.900	58.7%	61.6%
BMI	0.598	0.557–0.639	26.33	50.2%	64.1%
LDL-C	0.538	0.496–0.581	3.740	29.7%	79.7%
FBG	0.494	0.454–0.534	7.070	75.3%	30.7%
HDL-C	0.489	0.448–0.529	2.030	1.9%	99.0%

**Table 5:** Pairwise comparisons of the discriminative performance of GLM7 with individual metabolic parameters.

	ΔAUC	DeLong *P*	Holm-adjusted *P*
GLM7 vs TyG	+0.0058	0.668	1.000
GLM7 vs FBG	+0.1051	<0.001	<0.001
GLM7 vs TG	−0.0239	0.106	0.319
GLM7 vs HDL-C	+0.1105	0.0010	0.005
GLM7 vs LDL-C	+0.0610	0.013	0.053
GLM7 vs BMI	+0.0010	0.965	1.000

The clinical model included age, sex, BMI, smoking, alcohol consumption, hypertension, HbA1c, serum creatinine, ALT, AST, and eGFR. The likelihood-ratio *P* values were derived from likelihood-ratio tests comparing each extended model with the corresponding clinical model. DeLong *P* values were obtained using the DeLong test for paired ROC curves to compare the AUCs between models.

### 3.5 Incremental value of TyG and GLM7

To further evaluate the comparative performance and incremental discriminatory value of GLM7, we compared its discriminative performance with that of TyG, and further assessed the improvement in predictive capacity when GLM7 was incorporated into a conventional clinical prediction model (**Table 6**). The AUC of GLM7 was 0.599, and the AUC of TyG was 0.593, with no statistically significant difference observed between the two indices (DeLong test, *P* = 0.668). The AUC of the conventional clinical model was 0.754 (95% CI: 0.719–0.789). Incorporating TyG into the model increased the AUC to 0.760 (95% CI: 0.725–0.794), while incorporating GLM7 increased the AUC to 0.757 (95% CI: 0.723–0.792). Neither incorporation yielded a statistically significant improvement in AUC relative to the base clinical model (DeLong test, *P* = 0.218 for TyG and *P* = 0.438 for GLM7, respectively). However, likelihood ratio tests demonstrated that model fit was significantly improved after adding either TyG (χ²=7.43, *P* = 0.006) or GLM7 (χ²=5.70, *P* = 0.017).

**Table 6:** Incremental value of TyG and GLM7 beyond the clinical model for hyperuricemia.

Model	AUC	95% CI	DeLong *P*	Likelihood-ratio *P*
Clinical model	0.754	0.719–0.789	—	—
Clinical model + TyG	0.760	0.725–0.794	0.218	0.006
Clinical model + GLM7	0.757	0.723–0.792	0.438	0.017

ΔAUC represents the difference in AUC between GLM7 and the comparator variable (GLM7 minus comparator). *P* values were calculated using the DeLong test for paired ROC curves. *P* values were adjusted for multiple comparisons using the Holm method.

### 3.6 Sensitivity analysis

Spearman correlation analysis revealed that serum creatinine (SCr) exhibited a moderate-to-strong negative correlation with estimated glomerular filtration rate (eGFR) (ρ = −0.637, *P* < 0.001). In the fully adjusted regression model, the variance inflation factors (VIFs) for SCr and eGFR were 2.89 and 3.06, respectively. Based on the predetermined threshold of VIF < 5, the model did not present notable multicollinearity. Sensitivity analysis demonstrated that the positive association between GLM7 and hyperuricemia remained consistent when SCr or eGFR was included in the model separately (OR = 1.760, 95% CI: 1.334–2.321; OR = 1.813, 95% CI: 1.383–2.375, respectively; both *P* < 0.001). These results were consistent with the estimates from the model containing both SCr and eGFR (OR = 1.718, 95% CI: 1.294–2.282, *P* < 0.001).

## 4 Discussion

In this retrospective cross-sectional study enrolling 959 hospitalized patients with T2DM, we explored the correlation between the GLM7 and HUA. The main conclusions of the study are summarized as follows: First, GLM7 levels were significantly higher in T2DM patients with HUA compared with those without HUA. Second, after adjusting for demographic characteristics, lifestyle factors, glycemic control status, and renal and hepatic function indicators, GLM7 still remained an independent positive influencing factor for HUA. In the fully adjusted model, each 1-unit increment in GLM7 was associated with elevated odds of HUA (OR = 1.72, 95% CI: 1.29–2.28), and participants in the highest GLM7 quartile had higher odds of HUA than those in the lowest quartile (OR = 2.04, 95% CI: 1.28–3.26). Third, GLM7 was also positively correlated with serum uric acid (SUA) level: each 1-unit increment in GLM7 corresponded to a 37.88 μmol/L elevation in SUA level (95% CI: 28.22–47.55).

The above association remained consistent across all the clinical subgroups investigated in this study, and restricted cubic spline analysis confirmed a significant overall linear association between GLM7 and the risk of HUA, with no statistically significant non-linear relationship detected. In summary, our findings indicate that higher GLM7 is associated with higher risk of HUA and elevated SUA level in patients with established T2DM. However, limited by the cross-sectional study design, the observed results only reflect the correlation between variables rather than causal association.

The findings of the present study expand the limited existing clinical evidence on GLM7. GLM7 was initially developed based on NHANES data as a composite index that integrates seven routinely measured metabolic indicators, including age, BMI, fasting blood glucose, insulin, triglyceride, LDL-C and HDL-C, with the core objective of combining multiple routinely tested metabolic characteristics into a unified single indicator (14). To date, a small number of follow-up studies have explored the correlation between GLM7 and multiple metabolic disorders as well as chronic diseases, but the relevant research on the association between GLM7 and HUA, especially in patients with confirmed T2DM, is still insufficient. The results of the present study are consistent with the conclusions of the existing mainstream academic literature, which confirms that HUA often co-occurs with multiple metabolic abnormalities, including obesity, dyslipidemia, insulin resistance and diabetes (9, 10). Previous prospective studies and meta-analyses have further documented that elevated SUA level is correlated with an increased risk of subsequent T2DM onset, which supports the existence of a close epidemiological association between glucose metabolism and urate metabolism (11, 12). The present study extends the investigation of this association from the perspective of a composite glucolipid metabolic index, and demonstrates that the overall metabolic profile characterized by GLM7 is significantly associated with HUA among the population already diagnosed with T2DM.

A critical scientific question that needs to be addressed is whether the relatively complex GLM7 can provide additional explanatory information beyond the existing simpler metabolic indicators. Accordingly, this study compared the discriminatory performance of GLM7 with that of the triglyceride-glucose (TyG) index and each individual metabolic parameter. The results showed that the area under the receiver operating characteristic curve (AUC) of GLM7 was 0.599 (95% CI: 0.559–0.640), and there was no statistically significant difference between this value and the AUC of TyG, which was 0.593 (95% CI: 0.554–0.633; DeLong test, *P* = 0.668). Similarly, no statistically significant difference in discriminatory performance was detected between GLM7 and TG or between GLM7 and BMI. These findings are of reference value because the correlation between TyG and SUA as well as HUA in T2DM patients has been confirmed in previous studies. Specifically, in a Chinese study enrolling 719 non-obese patients with T2DM, TyG was confirmed to be independently associated with HUA, with an AUROC of 0.613 (18). Based on the above results, even though GLM7 integrates more metabolic components, it did not exhibit superior standalone discriminatory capacity relative to TyG in the study population. This study further explored whether GLM7 can provide incremental predictive information beyond conventional clinical variables. The results showed that incorporating GLM7 into the basic clinical model increased the AUC from 0.754 to 0.757, but this increment did not reach statistical significance (DeLong test, *P* = 0.438). Although the likelihood ratio test indicated that the addition of GLM7 significantly improved the model goodness of fit (χ² = 5.70, *P* = 0.017), the non-significant improvement in AUC suggests that this statistically significant improvement cannot be interpreted as evidence of clinically meaningful improvement in discriminatory performance. In summary, the findings of this study confirm the existence of a significant association between GLM7 and HUA, but do not support that GLM7 has superior clinical discriminatory performance compared with established metabolic indicators or conventional clinical information.

When evaluating the performance of GLM7 in patients with diagnosed T2DM, the incorporation of fasting blood glucose (FBG) as a constituent of this composite index requires special discussion. FBG is one of the seven core components that constitute the GLM7 indicator (14) and FBG itself is also a widely recognized biochemical indicator for diabetes diagnosis. Therefore, when GLM7 is applied to the diabetic population, given that one of its core components is inherently associated with the definition and metabolic characteristics of diabetes, the absolute distribution of GLM7 in this population will shift toward higher values relative to the general population. This distribution shift may restrict the direct extrapolation of GLM7 thresholds or performance estimation results obtained from populations with different diabetes prevalence or glycemic characteristics. This conceptual overlap between FBG and diabetes status has also been emphasized in recent methodological discussions targeting the original GLM7 research (19, 20).

Notably, the present study specifically assessed GLM7 in a confirmed diabetic population, rather than aiming to discriminate between diabetic and non-diabetic individuals. In the study cohort, no statistically significant difference in FBG level was identified between patients with and without HUA, while GLM7 level remained significantly higher in the HUA group, and GLM7 was independently correlated with HUA after multivariable adjustment. This result indicates that the association detected in this study cannot be simply attributed to the higher FBG concentration in HUA patients. Nevertheless, due to the incorporation of FBG in its calculation framework, GLM7 cannot be considered completely independent of glycemic status. Accordingly, the results of this study should be interpreted as evidence that the comprehensive metabolic profile characterized by GLM7 is associated with HUA in patients with established T2DM, rather than evidence that GLM7 can provide a measurement of HUA that is completely independent of glycemic status. This distinction is of great significance for the clinical interpretation of GLM7 in diabetic populations. Future research is needed to verify whether the index still maintains similar associations after modification or recalibration of its glycemic component, and whether its predictive performance differs between diabetic and non-diabetic populations.

Beyond renal urate excretion, hepatic urate production may also contribute to the relationship between metabolic dysregulation and hyperuricemia. Previous experimental research has demonstrated that fatty acid oxidation may promote hepatic urate synthesis through HIF-1α activation and subsequent upregulation of NT5C2 and XDH (21).

Renal function constitutes a critical determinant of SUA homeostasis, as impaired glomerular filtration capacity and dysregulated tubular urate transport mechanisms collectively reduce urate clearance efficiency. In the present study, the association between GLM7 and HUA remained statistically robust in sensitivity analyses adjusting for serum creatinine and eGFR, both individually and concurrently. These findings indicate that the observed association is not substantially confounded by the intrinsic correlation between GLM7 and these two renal function biomarkers. It should be explicitly emphasized that the mechanistic hypotheses proposed herein are purely speculative rather than definitive conclusions verified by empirical evidence from this study, as the current research design did not incorporate direct measurements of urinary urate excretion, fractional urate excretion, urate transporter expression levels, or hepatic urate production kinetics.

Dose-response analysis and subgroup stratification provide additional contextual support for the interpretation of the observed association. Restricted cubic spline analysis demonstrated a statistically significant overall association between GLM7 and HUA, while no statistically significant evidence of nonlinearity was detected across the observed range of GLM7 values. Accordingly, the current dataset does not support the presence of a notable deviation from an approximately linear dose-response relationship within the studied GLM7 interval. This observation is consistent with the progressive elevation in HUA odds observed across increasing GLM7 quartiles in the primary analysis. Nevertheless, the absence of statistically significant nonlinearity cannot be interpreted as definitive evidence for a strictly linear biological association or a causal dose-response relationship. Similarly, the positive association between GLM7 and HUA remained directionally consistent across all strata defined by age, sex, BMI, hypertension status, smoking status, and alcohol consumption, with no statistically significant interaction effects identified. These findings indicate that the observed association is not restricted to a specific clinically defined subgroup; however, further large-scale, population-based studies are warranted to confirm whether clinically meaningful effect modification exists in broader patient populations.

Interpretation of the present study findings necessitates careful consideration of the enrolled cohort characteristics. The male predominance observed in this hospitalized cohort reflects the patient population of this single tertiary medical center, rather than a deliberate sex-matched sampling framework. Although sex was incorporated as a covariate in the multivariable regression analyses and no statistically significant sex interaction was identified, this imbalanced sex distribution may limit the generalizability of the study findings to populations with distinct demographic compositions. Furthermore, this study exclusively recruited hospitalized patients with established T2DM, who typically exhibit more severe metabolic dysregulation compared with community-dwelling diabetic individuals. Accordingly, the observed association cannot be directly extrapolated to the broader Chinese diabetic population in the absence of external validation.

The clinical implications of the present study findings warrant cautious interpretation. Although GLM7 is independently and positively associated with HUA and SUA levels, its independent discriminatory capacity remains limited: the area under the receiver operating characteristic curve (AUC) is 0.599 (95% CI: 0.559–0.640), with an optimal cutoff value of 7.959 corresponding to a sensitivity of 63.3% and specificity of 55.0%. Moreover, the incorporation of GLM7 into the conventional clinical prediction model did not yield a statistically significant improvement in model AUC. Consequently, the study results do not support the utilization of GLM7 as a standalone diagnostic or screening instrument for HUA, nor do they validate GLM7 as a superior risk stratification index. Instead, GLM7 may serve as a composite metabolic indicator reflecting the overall glucolipid metabolic status correlated with HUA in patients with T2DM. Direct measurement of SUA remains an indispensable component of HUA diagnostic protocols. The coexistence of a statistically significant epidemiological association and limited discriminatory performance further underscores the principle that an observed association does not inherently confer sufficient predictive accuracy for routine clinical application.

Another unresolved question relates to the population generalizability of the GLM7 index. The original GLM7 index was constructed based on NHANES data derived from a US population, and has only been evaluated in external datasets thereafter (14). The present study only conducted a preliminary analysis of the association between GLM7 and HUA in the Chinese population with confirmed T2DM, and did not complete a systematic external validation of the original GLM7 index. Variations in population demographic characteristics, dietary structure, lifestyle habits, medical service systems, metabolic phenotypes and clinical diagnostic criteria may all influence the distribution and predictive efficacy of composite metabolic indices. Accordingly, the results of this study should be understood as population-specific association evidence, rather than conclusive proof that the original GLM7 model maintains consistent predictive performance in Chinese T2DM patients. Future research based on independent multi-center cohorts and standardized harmonized external datasets (including the NHANES dataset when conditions permit) is required to verify whether the observed association in this study is replicable, and to clarify whether it is necessary to perform recalibration or population-specific adaptive modification for the GLM7 index.

## 5 Limitations

This study still has several other limitations. First, the retrospective cross-sectional study design adopted in this research cannot support inference on the temporal order of exposure and outcome, nor can it draw causal conclusions; meanwhile, the possibility of reverse causation cannot be ruled out. Second, the study sample was recruited from a single-center population of hospitalized patients, which may introduce selection bias and limit the generalizability of the research conclusions. Third, this study did not collect detailed data on dietary purine intake, fructose intake, physical activity, and actual alcohol intake, so the possibility of residual confounding cannot be excluded. Fourth, the study did not obtain direct measurements of urinary urate excretion, fractional urate excretion, hepatic urate production, and the activity of renal and intestinal urate transporters. Accordingly, this study cannot clarify whether the observed association is primarily caused by increased urate production, impaired renal urate excretion, altered intestinal urate excretion, or the combined effect of multiple mechanisms. Finally, although the inclusion of GLM7 improved model fitness according to the likelihood ratio test, it did not significantly improve the area under the receiver operating characteristic curve (AUC), and the standalone AUC of the GLM7 model remained at a moderate level. Therefore, before GLM7 can be applied clinically for hyperuricemia-related risk assessment, prospective multi-center studies with direct detection of urate metabolism indicators and independent external validation are still needed.

## 6 Conclusion

In conclusion, the present study confirms that GLM7 is independently and positively correlated with both HUA and SUA levels among hospitalized patients with T2DM. This correlation remains consistent across all the clinical subgroups analyzed in this study, and no significant non-linear correlation is detected. Nevertheless, GLM7 does not exhibit superior discriminative performance relative to the TyG index, and integrating GLM7 into a conventional clinical prediction model does not significantly improve the area under the receiver operating characteristic curve (AUC). In addition, the inclusion of FBG as a component of GLM7 requires special attention when interpreting the index in populations with confirmed diabetes, since this component may lead to a systematic upward bias in GLM7 values and restrict direct comparisons with thresholds established for the general population. Collectively, the results of this study support that GLM7 can act as a composite metabolic marker associated with HUA in T2DM populations, but this study does not verify that GLM7 can be used as a diagnostic, screening or risk stratification tool. Additional prospective studies and external validation are required to further clarify the generalizability of GLM7 and its potential clinical application value.

## Data Availability

The raw data supporting the conclusions of this article will be made available by the authors, without undue reservation.
